# Development and psychometric evaluation of a new brief scale to measure eHealth literacy in people with type 2 diabetes

**DOI:** 10.1186/s12912-022-01062-2

**Published:** 2022-11-04

**Authors:** Eun-Hyun Lee, Young Whee Lee, Kwan-Woo Lee, Hae Jin Kim, Seongbin Hong, So Hun Kim, Eun Hee Kang

**Affiliations:** 1 Graduate School of Public Health, Ajou University, 164 Worldcup-ro, Yeongtong-gu, 16499 Suwon, Gyeonggi-do Republic of Korea; 2 Department of Nursing, Inha University, Incheon, Republic of Korea; 3 Department of Endocrinology and Metabolism, School of Medicine, Ajou University, Suwon, Republic of Korea; 4 Department of Internal Medicine, School of Medicine, Inha University, Incheon, Republic of Korea

**Keywords:** Diabetes, Electronic health literacy, Instrument, Psychometrics, Scale

## Abstract

**Background:**

The internet has become a major source of health information, and obtaining appropriate information requires various abilities and skills, labeled as electronic health literacy (eHealth literacy). The existing instruments for measuring eHealth literacy are outdated because they were developed during the Web 1.0 era, or not sufficiently sensitive for people with a specific condition or disease because they were designed to assess eHealth literacy over a broad range for a general population. Approximately one in ten adults worldwide live with diabetes. Health professionals have a responsibility to identify patients with low eHealth literacy to prevent them from obtaining misleading internet diabetes information.

**Aims:**

The aims were to develop a condition-specific eHealth literacy scale for diabetes and to evaluate its psychometric properties among people with type 2 diabetes.

**Methods:**

An instrument development design was used. This study recruited 453 people diagnosed with type 2 diabetes at the outpatient clinics of hospitals in 2021. Psychometric properties (internal consistency, measurement invariance, and content, structural, convergent, and known-groups validities) were analyzed.

**Results:**

An expert panel assessed content validity. Exploratory factor analysis, exploratory graph analysis, and confirmatory factor analysis (CFA) for structural validity yielded a two-factor solution (CFI = 0.977, SRMR = 0.029, RMSEA = 0.077). Cronbach’s alpha and omega values were excellent for each factor (0.87–0.94). Multigroup CFA yielded configural and metric measurement invariance across the gender, age, and glycemic control status groups. Convergent validity with a comparator instrument to measure health literacy was supported by a moderate correlation, and known-groups validity determined using groups with different internet-use frequencies was satisfied with a high effect size.

**Conclusion:**

A new condition-specific eHealth literacy scale for people with type 2 diabetes was developed, comprising 10 items. The scale exhibited good psychometric properties; however, test–retest reliability must be determined for the stability of the scale and cross-cultural validity is required among different languages. The brief scale has the merits of being feasible to use in busy clinical practice and being less burdensome to respondents. The scale can be applied in clinical trials of internet-based diabetes interventions for assessing the eHealth literacy of respondents.

**Supplementary Information:**

The online version contains supplementary material available at 10.1186/s12912-022-01062-2.

## Background

The internet has become a major source for obtaining information. There are currently 5.17 billion internet users worldwide, representing approximately 65.6% of the global population [1]. Based on world regions, North America has the highest internet usage rate of 93.9%, followed by Europe at 88.2% [1]. South Korea is the country with the highest internet penetration rate of 97% [2], and the rate of smartphone use among adults in the country was 95% [3]. With widespread access to the internet, individuals now use the internet for diverse purposes in their everyday lives. Using the internet to access health information is common, with one in two EU citizens (55%) aged 16–74 years looking for health information on the internet [4]. About 75.1% of adults in the US searched for health information on the internet each month, such as on diet/nutrition, exercises, medicines, and remedies [5].

Obtaining useful health information from the internet requires various abilities and skills, including not only the basic abilities of reading/writing and understanding health information, but also the skills of searching for and acquiring health information in the internet environment using digital devices. These abilities and skills were labeled together as electronic health literacy (eHealth literacy) in 2006, which was defined as “the ability to seek, find, understand, and appraise health information from electronic sources and apply the knowledge gained to address or solve a health problem” [6].

Several self-reporting instruments have been developed over the last 15 years to assess eHealth literacy. The eHealth Literacy Scale (eHEALS) was the first instrument developed [6], and has been psychometrically evaluated among 18 different languages and in 26 countries [7]. With the shift of information technology from Web 1.0 to Web 2.0, the pioneering instrument of the eHEALS developed in 2006 has not received sufficient attention regarding social media and mobile web skills [8, 9]. The need for an instrument that includes the attributes of skills required to use interactive communication technologies on the internet was reported by van der Vaart et al. [10].

With such a perspective, second-generation instruments were developed, including the e-Health Literacy Scale (e-HLS) [11], Digital Health Literacy Instrument (DHLI) [12], eHealth Literacy Assessment (eHLA) [13], eHealth Literacy Questionnaire (eHLQ) [14], and Transactional eHealth Literacy Instrument (TeHLI) [15]. However, the recent systematic review of measurement properties indicated that the second-generation instruments have considerable limitations [7]. Clearly defining the construct being measured is fundamental in developing a self-reporting instrument, but most of the second-generation instruments are not based on clear statements about what is being measured. This suggests the need for a new instrument in which the eHealth literacy definition being measured is clearly addressed. Further studies are also needed to increase the credibility of the psychometric evidence for these instruments, because the e-HLS and the TeHLI have insufficient low-quality evidence for convergent validity, the DHLI has sufficient low-quality evidence for structural validity, the eHLA has insufficient very-low-quality evidence for internal consistency, and the eHLQ has indeterminate low-quality evidence for measurement invariance [7].

When selecting a self-reporting instrument, the population in which the instrument is administered must be considered to determine whether to use a generic- or condition-specific instrument [16]. A generic instrument is designed to assess a construct (e.g., eHealth literacy) in a broad population, so that it can be applied to a healthy population, across healthy and disease populations, or across different disease populations. Its limitation is that it might not be sufficiently sensitive to assess the construct being measured in a population with a specific condition, because important attributes to the condition or disease are not fully covered. A condition-specific instrument focusing on the contents relevant to clinical conditions is recommended for use in a patient population [17].

The searching contents or abilities of people with a particular disease, which are associated with internet health information, were demonstrated to be different from other diseases. For example, Madrigal and Escoffery [5] found that people with a chronic disease were more likely to search for information on their medicines. The ability of patients with a particular chronic disease to process internet health information is different from those of patients with other diseases [18]. The condition-specific types of eHealth literacy instruments may therefore be a better fit for a patient population than generic types. The Transactional eHealth Literacy Instrument [15] is the only instrument developed in the specific populations of baby-boomer and older adults with chronic lung disease.

Diabetes is a global health problem, with approximately 537 million adults living with diabetes (1 in 10 adults), and about 90% of all diabetes cases being type 2 diabetes. [19]. It requires complex ongoing self-management in the daily lives of patients (e.g., dieting, physical exercise, monitoring blood glucose levels, administration of medications, foot care, and stress control). In the digital era, many people with diabetes obtain information on the disease and its self-management from the internet via digital devices, due to the reduced restriction from time and location [20]. Along with the shifting of diabetes information sources, eHealth literacy has emerged as an important determinant of enhancing diabetes self-management [21, 22]. In practice, many internet-based interventions for diabetes have been developed and serviced to empower patients to engage in self-management and control their glycemic levels [23–25]. It is essential for clinicians to assess the eHealth literacy levels of their patients when education or interventions are provided in the form of technology or digital services [26]. However, there is no condition-specific instrument that measures eHealth literacy specific to diabetes. The aims of this study were therefore to develop the Condition-Specific eHealth Literacy Scale for Diabetes (designated as the CeHLS-D) and to evaluate its psychometric properties.

## Methods

The CeHLS-D was developed and evaluated in four phases in this study: Conceptualization, Item generation, Content validation, and Field survey for quantitative psychometric tests.

### Phase I: conceptualization

The initial step of developing the new scale was to conceptualize the construct being measured, which considered a target population for whom the developed scale is intended for use [16]. Since the first definition of eHealth literacy by Norman and Skinner in 2006, many definitions have been introduced, but without consensus. Griebel et al. [8] recently defined eHealth literacy as “a dynamic and context-specific set of individual and social factors as well as technology constraints in the use of digital technologies to search, acquire, comprehend, appraise, communicate, apply and create health information in all contexts of healthcare with the goal of maintaining or improving the quality of life throughout the lifespan” (p. 433), mostly based on the meta-definition proposed by Bautista [27], but with additional aspects from the definitions of others [28, 29].

The target population for the scale developed in the present study was adults diagnosed with type 2 diabetes. This group encounters or needs health information specific to their disease, treatment, and complex self-management to prevent the onset and progression of complications and to improve quality of life [30, 31]. Based on those perspectives, eHealth literacy was conceptualized in the present study as the abilities and skills to search, acquire, comprehend, appraise, communicate, apply, and create health information specific to diabetes, and its treatment and self-management in internet environments using digital devices, with the goals of improving or maintaining health and preventing complications to improve health-related quality of life. Internet environments in the present study not only refer to the read-only mode of the web but also to participative social media. The digital devices considered included personal computers, mobile phones, and tablets.

### Phase II: item generation

For item generation during the development of the new scale, it was important to pool all attributes reflecting the construct being measured. A literature review and a semistructured interview were used as the sources of the attributes in this study. For the comprehensive literature review, a matrix table was constructed based on the above-mentioned eHealth literacy conceptualization. The top row of the matrix contained posited abilities and skills (search, acquire, comprehend, appraise, communicate, apply, and create). In the left column of the matrix, internet environments were posited: static searching portal (e.g., Google and NAVER), email/mobile text messengers (e.g., Gmail, NAVER Mail, KakaoTalk, and WhatsApp), and social network/media sharing (e.g., Facebook, Twitter, and YouTube). From the literature review, the cells of the matrix constructed by overlapping columns and rows were filled with the attributes regarding information on diabetes, and its treatment and self-management.

A semistructured interview was conducted by a trained interviewer (a nursing PhD candidate) in a small room at an outpatient clinic in June 2021. The inclusion criteria for the participants were being at least 19 years old, diagnosed with type 2 diabetes, and an internet user. The appropriate sample size in a qualitative interview is determined by data (attributes) saturation, referring to when collecting more data no longer yields any new data. In this study the interview initially included 20 participants, which is a commonly recommended sample size for research involving qualitative interviews [32]. Those who agreed to participate in the interview were asked to sign an informed-consent form. Each interview was conducted based on the above matrix table, and was recorded and transcribed verbatim. One researcher presented the eHealth literacy-related attributes by using the actual words said by the interviewees and filled in the matrix table. These processes were confirmed and discussed with another expert on eHealth and diabetes care.

### Phase III: content validity

#### Expert panel

Content validity refers to the degree to which each item reflects the construct being measured [33]. A panel of five experts on eHealth literacy, measurement properties, and diabetes care were participated in the content validity. They were asked to respond on how much relevant each item was using a four-point Likert scale (1 = “not relevant,” 2 = “somewhat relevant,” 3 = “quite relevant,” and 4 = “very relevant”).

#### Analysis of content validity

Content validity was assessed using the item-level content validity index (I-CVI) [34]. The I-CVI was calculated as the proportion of experts who answered “quite relevant” or “very relevant.” If I-CVI > 0.78, the item was considered sufficiently relevant to the eHealth literacy construct. Open questions were also asked to the expert panel to ascertain comprehensiveness (if any of the key construct aspects were missed), comprehensibility (reading level, jargon, and ambiguity), an item response format with a five-point Likert scale ranging from 0 (“not at all”) to 4 (“very much”), and instructions on how to respond to items.

### Phase IV: field survey

#### Study design

A cross-sectional survey was conducted to evaluate the internal consistency, measurement invariance, and structural, convergent, and known-groups validities of the CeHLS-D.

#### Sample and data collection

A convenience sample of 453 participants was recruited from outpatient clinics in multiple hospitals in South Korea from August to December in 2021. The inclusion criteria for the sample were being at least 19 years old, diagnosed with type 2 diabetes, experienced in using digital devices (personal computers, mobile phones, or tablets), and articulate in the Korean language. Trained research assistants met and provided the study information to potential participants at outpatient clinics. Those who agreed to participate in this study were asked to sign an informed-consent form and then to complete questionnaires. All participants were offered remuneration for participation.

#### Measures

For convergent validity, eHealth literacy was expected to be moderately correlated with health literacy, based on previous studies [35, 36]. The Diabetes Health Literacy Scale (DHLS) [30] was administered in this study as a comparator instrument to assess the convergent validity of the CeHLS-D. The DHLS was developed to measure diabetes-specific health literacy, and comprises 14 items scored on a 5-point Likert scale from 0 to 4. The scale score is the average of all items, with higher scores indicating better health literacy. The DHLS yielded good psychometric properties for content validity, structural validity (χ^2^/*df* = 2.41, RMSEA = 0.07, SRMR = 0.04, and CFI = 0.95), convergent validity, criterion validity, internal consistency (Cronbach’s alpha = 0.91), and test–retest reliability (intraclass correlation coefficient = 0.89). Cronbach’s alpha of the scale in the present study was 0.94. Cronbach’s alpha of the scale in the present study was 0.94.

The following question was asked about the frequency of internet use: “How often do you use the internet to seek health information?” There were four response options of “almost no use,” “approximately 1 day a week,” “several days a week,” and “almost every day.” This was administered to assess the known-groups validity of the CeHLS-D, because people who use the internet more frequently have a higher eHealth literacy than those who use it less [37]. If the mean CeHLS-D score increased with the frequency of internet use, the scale was considered to have satisfactory known-groups validity.

According to the systematic review of existing eHealth literacy instruments [7], a few measurement invariances were tested across groups, including demographic (gender and age), cultural, and physical activity frequency groups. Similarly, the measurement invariances in gender, age, and glycemic control status in the CeHLS-D were presented: male vs. female, ≥ 60 vs. < 60 years old, and glycated hemoglobin A1c (HbA1c) ≤ 6.5% vs. HbA1c > 6.5%. HbA1c values were collected from medical records from within the previous 3 months.

### Data analysis

The data were analyzed using SPSS for Windows (version 25), AMOS software (version 25), and the R statistical environment [38]. Missing data were replaced using regression imputation. Mean and standard-deviation values of the items were computed using descriptive statistics. An interitem correlation matrix of all items was conducted, and weakly correlated (*r* < .30) or redundant (*r* > .80) items were removed [39].

For the cross-validation of structural validity, the total sample was split into two subsamples using the SPSS random assignment function. Subsample 1 (*n* = 231) was used for exploratory factor analysis (EFA) and exploratory graph analysis (EGA), and subsample 2 (*n* = 222) was used for confirmatory factor analysis (CFA). The sample size of each subsample satisfied 7 times the number of items for EFA and at least 200 cases for CFA [40, 41].

To determine whether the application of EFA to the subsample 1 data was available, the Kaiser-Meyer-Olkin (KMO) test and Bartlett’s test of sphericity were conducted [42]. EFA with varimax rotation was conducted to reduce the number of items and determine their underlying structure. Factors with eigenvalue > 1 were retained, and the results was satisfactory when at least 50–60% of the variance was explained by the factors [39]. Factor loadings higher than 0.70 were considered significant to capture the essence of a factor [16]. The dimensionality and patterns of items clustered together in the EFA were further assessed using EGA, which is a new approach for identifying the dimensions of constructs based on network psychometrics [43]. The EGA involves depicting a network as nodes (test items) that are connected by edges (links) representing the internode strengths (i.e., partial correlations). The EGA was conducted using a graphical least absolute shrinkage and selection operator from the EGAnet package.

CFA was performed on subsample 2 using maximum-likelihood estimation. The CFA model fit was determined using multiple indices: normed χ^2^ (χ^2^/*df* < 3), comparative fit index (CFI) > 0.95, standardized root-mean-square residual (SRMR) < 0.08, and root-mean-square error of approximation (RMSEA) < 0.08 [44]. Supplementary to the CFA, the heterotrait-monotrait ratio of correlations (HTMT) was calculated to determine whether a pair of factors (subscales) derived by CFA was distinctively different from another [45]. An HTMT of < 0.85 suggested that the pair of factors was discriminant [46].

For internal consistency analysis, traditional Cronbach’s alpha was assessed, with acceptable values ranging from 0.70 to 0.95 [47]. In a more robust manner, McDonald’s omega (ω) was computed with the criterion value of > 0.70 [48].

Measurement invariance across the gender, age, and glycemic control status groups were analyzed using multigroup CFA (MGCFA). Sample sizes of at least 100 in each of the gender, age, and glycemic control status groups were satisfied for the MGCFA [46]. The MGCFA was tested in the following successive phases using AMOS software [49]: configural invariance model (a baseline model for comparing subsequent invariance tests), metric invariance model (all factor loadings were constrained equally, which is called the measurement weights model), structural covariances model (factor loadings, factor variances, and covariances were constrained equally), and measurement residuals model (factor loadings, factor variances, factor covariances, and error variances were constrained equally). The first two models were given the most attention in practice since the others were considered excessively stringent tests that often are not satisfactory [50]. Configural and metric invariance models were therefore tested in the present study. A CFI change ($\varDelta$CFI) of <–0.010, supplemented by either an RMSEA change ($\varDelta$RMSEA) of < 0.015 or an SRMR change ($\varDelta$SRMR) of < 0.030, indicated invariance on the metric invariance model test [51]. A χ^2^ difference test is a traditional method for measurement invariance decisions on criteria, but has the limitation of being sensitive to a large sample [49], and was therefore not used in this study.

Convergent validity was analyzed using Pearson’s correlation coefficient. Known-groups validity was tested using one-way analysis of variance (ANOVA). The magnitude of known-groups validity was assessed using the effect size of an eta-squared value (*η*^2^), with values of 0.01, 0.06, and 0.14 indicating small, moderate, and large effects, respectively [52].

The floor and ceiling effects of the potential scores were explored using descriptive statistics, and interpreted if 15% or more of the respondents achieved the lowest and highest scores on the instrument [53].

## Results

### Items derived

The literature review extracted an initial pool of 28 attributes that filled the cells of the matrix table constructed in this study (more than one attribute was allowed in a cell). Each semistructured interview lasted about 30 min. Attributes saturation occurred at the 14th participant in this study. This is consistent with a recent systematic review finding that saturation occurred after 9–17 interviews in qualitative research [54]. General characteristics are listed in Table S1 in the Supplementary Material. From the semistructured interview, the following four additional attributes were added to the initial pool of attributes: thinking of appropriate search words, blocking out spammers, distinguishing whether a text message (e.g., information for visiting a clinic or receiving a medical examination) is for someone or anyone, and protecting personal information. Each attribute on the matrix table was then converted into the content of each item.

### Content validity

Of the derived items, 29 achieved I-CVI > 0.78, and the remaining items not satisfying the criterion value were deleted. As suggested by the expert panel, some phrases of five items were slightly modified to increase comprehensibility, but no new items were added. The five-point Likert scale was unchanged. The instructions on how to respond to items was maintained in which the asking recall period was “at the present time.” The content-validated items were then checked by a professional who majored in the Korean language, and seven items were semantically polished into plain language.

### Field survey

#### General characteristics

Among the 453 participants, more than a half were male (64.7%), employed (66.2%), and had graduated from high school (87.9%). They were aged 56.8 (SD = 10.8) years. Approximately two-thirds of the participants were taking an oral hypoglycemic agent (78.1%), and their diabetes duration was 8.9 (SD = 7.3) years (Table S2 in the Supplementary Material).

#### Interitem correlation matrix

The interitem correlation coefficients of all items ranged from 0.42 to 0.89 (*p* < .05). Ten item pairs were strongly correlated (coefficient > 0.80). One item from each strongly correlated pair was removed because they indicated item redundancy, leading to a multicollinearity problem [39].

#### Structural validity

**Fig. 1:**
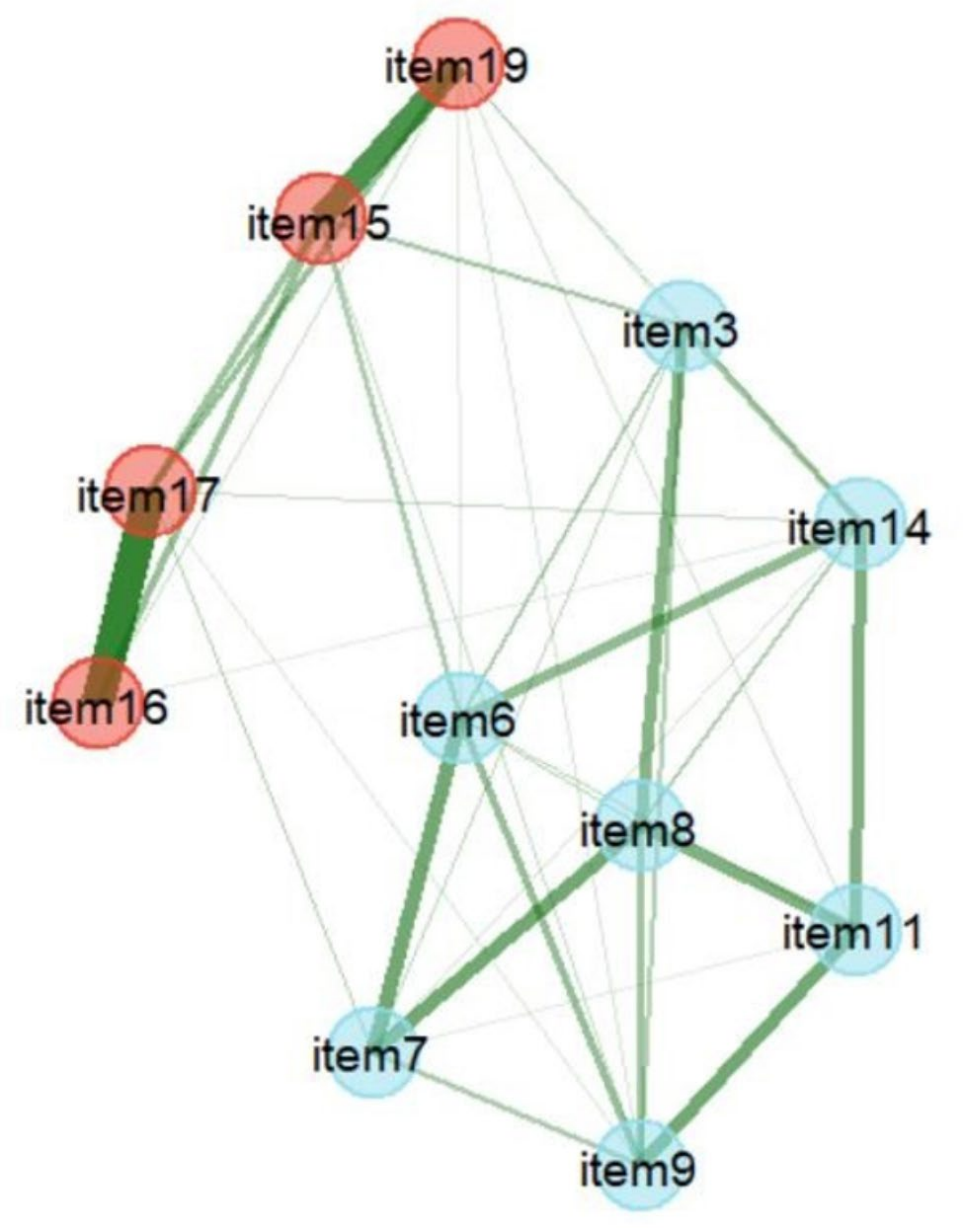
Exploratory graph analysis (EGA) of the number of factors

EFA was conducted on subsample 1 (Table 1) Bartlett’s test was significant (χ^2^ = 4242.46, *p*$<$0.001) and KMO = 0.95, implying that the data had very good factorability. EFA with varimax rotation extracted a two-factor solution (eigenvalue > 1), and the amount of variance explained by the two factors was 70.00%. A total of 12 items were meaningfully loaded onto one of the 2 factors. Factors 1 and 2 were loaded with eight and four items, respectively. If Cronbach’s alpha was > 0.95, some items in the factor might be redundant [47]. Cronbach’s alpha of factor 1 was > 0.95. Item 10 was also very strongly correlated with the total score of factor 1 (a corrected item-total correlation of *r* = .91) implying the existence of a redundant item [39], and so it was deleted. The second EFA was then performed with 11 items, and extracted a 2-factor solution explaining 75.55% of the variance (Table 1). Factors 1 and 2 were named “cognitive actions for internet diabetes information” and “abilities of digital communication,” respectively. The EGA also demonstrated two dimensions, as depicted in Fig. 1. The patterns of items clustered together were consistent with the EFA results, and the partial correlation between items 16 and 17 was the strongest.

**Table 1:** Scores for each item and findings of exploratory factor analysis (EFA)

			First EFA^b^		Second EFA^c^	
No.	Abbreviated item description^a^	Mean (SD)	Factor 1	Factor 2	Factor 1	Factor 2
1	Finding questions on the internet	2.58 (1.29)				
2	Preference of searching the internet	2.71 (1.26)				
3	Thinking of search words	2.46 (1.22)	0.73		0.73	
4	Navigating websites	2.40 (1.27)				
5	Storing information	2.22 (1.50)				
6	Understanding medical terms	2.35 (1.24)	0.77		0.80	
7	Figuring out numeric medical examination values (e.g., HbA1c, fasting glucose)	2.57 (1.16)	0.78		0.79	
8	Appraising information credibility	2.28 (1.15)	0.85		0.88	
9	Distinguishing from advertisements	2.38 (1.18)	0.79		0.81	
10	Judging a website to be sought	2.42 (1.15)	0.86			
11	Trustworthiness of internet sources	2.09 (1.15)	0.80		0.83	
12	Questioning and answering on a website	1.92 (1.22)				
13	Presearching before seeing health professionals	1.93 (1.30)				
14	Filtering applicable information	2.09 (1.56)	0.72		0.75	
15	Emailing	2.50 (1.56)		0.76		0.75
16	Text messaging (e.g., KakaoTalk^d^, WhatsApp^d^)	3.19 (1.04)		0.85		0.89
17	Attaching a file to a text message	3.07 (1.17)		0.82		0.87
18	Credibility of social media information	2.28 (1.18)				
19	Sharing opinions on social media (e.g., Instagram, YouTube)	2.10 (1.46)		0.77		0.73
Percentage of variance explained			70.00%		75.55%	
Cronbach’s alpha			0.952	0.89	0.94	0.89
Omega ($\omega$)			0.952	0.88	0.94	0.88

HbA1c, glycated hemoglobin A1c; SD, standard deviation

^a^ Diabetes- and self-management-related internet information and sources

^b^ Kaiser-Meyer-Olkin = 0.95, Bartlett’s χ^2^ = 4242.46 (*p* < .001)

^c^ Kaiser-Meyer-Olkin = 0.92, Bartlett’s χ^2^ = 2155.85 (*p* < .001)

^d^ A free mobile instant text messaging app

Blue and red nodes indicate factors 1 and 2, respectively. Items are numbered as in Table 1. Line thicknesses indicate the strength of partial correlations.

**Table 2:** Summary of fit indices in confirmatory factor analysis (CFA)

Model	χ^2^	*df*	χ^2^/*df*	CFI	SRMR	RMSEA (90% CI)	$\varDelta$CFI
Initial model	169.559*	43	3.943	0.945	0.074	0.115 (0.097–0.134)	NA
Modified model 1^a^	87.464*	42	2.082	0.980	0.030	0.070 (0.049–0.091)	0.035
Modified model 2^b^	78.502*	34	2.309	0.977	0.029	0.077 (0.055–0.099)	0.032

*df*, degrees of freedom; CFI, comparative fit index; SRMR, standardized root-mean-square residual; RMSEA, root-mean-square error of approximation; $\varDelta$CFI, change in CFI compared with the initial model; NA, not available; CI, confidence interval

^a^ Covariance between the measurement errors of items 16 and 17

^b^ Model after eliminating item 17

* *p* < .01

**Fig. 2:**
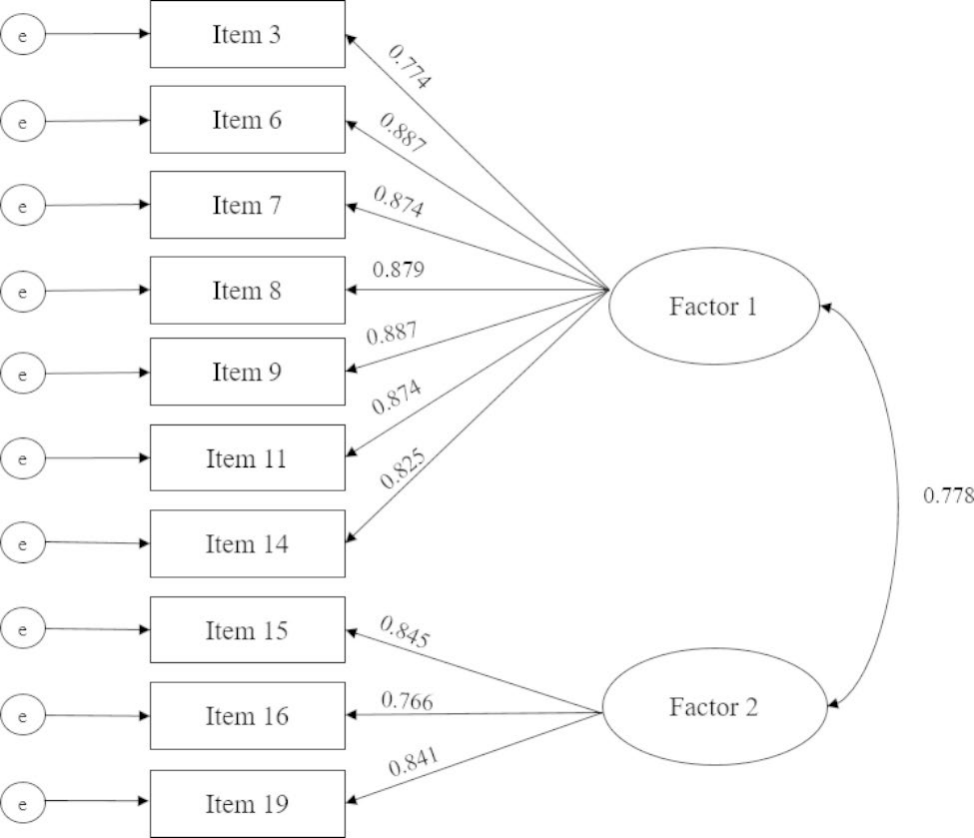
Findings of confirmatory factor analysis for the CeHLS-D

Based on the EFA/EGA results, CFA was performed on subsample 2 using the two-factor model. As presented in Table 2, the initial two-factor model provided a marginal fit to the data. The possibility of model misspecification was therefore explored: the modification index value was the highest between the error terms of items 16 and 17 (46.07) in factor 2. The two error terms of the items had their covariance presented with two-headed curved arrows, and CFA was again performed. This modification (modified model 1) markedly improved the fit ($\varDelta$χ^2^ [1] = 82.05, *p* < .05, $\varDelta$CFI = 0.035), and the values of the model-fit indices were satisfied (Table 2). However, the standardized error covariance parameter estimate between items 16 and 17 was somewhat high (0.745). This implies overlap in the content of the items, although they were worded differently but asked the same question [49]. Both items 16 and 17 related to “the skills about text messages.” After eliminating item 17, modified model 2 represented a meaningful improvement over the initial model ($\varDelta$CFI = 0.032) and yielded a good fit across all indices (Table 2). All of the loaded items were significant in their designated factors (critical ratio value > 1.96), and standardized factor loading values ranged from 0.766 to 0.887. The standardized factor covariance parameter estimate ($\phi$) was 0.778 (Fig. 2). HTMT was 0.76 (the criterion value was < 0.85), hence satisfying that the discriminant structure of the two factors.

Factor 1, cognitive actions for internet diabetes information; Factor 2, abilities of digital communication; e, measurement error. Items are numbered as in Table 1.

#### Internal consistency

For the total sample, Cronbach’s alpha values of factors 1 and 2 were 0.92 and 0.89, respectively. The $\omega$ values of factors 1 and 2 were 0.94 and 0.87, respectively. The CeHLS-D therefore demonstrated excellent internal consistency.

#### Measurement invariance

Table 3 presents the nested tests of measurement invariance for the CeHLS-D across the gender, age, and glycemic control status groups. All of the configural invariance models exhibited a satisfactory fit to the data. All of the metric invariance models displayed satisfactory fit to the data, and the $\varDelta$CFA, $\varDelta$SRMR, and $\varDelta$RMSEA values satisfied their invariance criteria. The measurement invariance of the CeHLS-D was therefore supported.

**Table 3:** Multiple indices for the measurement invariance of the CeHLS-D across the sex, age, and glycemic control status groups

Invariance model	χ^2^	***df***	CFI	SRMR	RMSEA	$\varDelta$CFI	$\varDelta$SRMR	$\varDelta$RMSEA
Gender								
Configural	156.860*	68	0.976	0.035	0.054	NA	NA	NA
Metric	164.465*	76	0.976	0.036	0.051	0.000	0.001	−0.003
Age								
Configural	163.453*	68	0.972	0.040	0.056	NA	NA	NA
Metric	185.904*	76	0.968	0.043	0.057	−0.004	0.003	0.001
Glycemic control status								
Configural	155.624*	68	0.976	0.038	0.053	NA	NA	NA
Metric	157.983*	76	0.978	0.038	0.049	0.002	0.000	−0.004

CFI = comparative fit index; *df* = degrees of freedom; SRMR = standardized root mean square residual; RMSEA = root mean square error of approximation; $\varDelta$: change in the value compared with that in the configural model; NA: not available

* *p* < .001

#### Convergent and known-groups validities

The CeHLS-D had a moderate correlation with the DHLS (*r* = .57, *p* < .001), suggesting that convergent validity was satisfied. One-way ANOVA revealed statistically significant differences in the mean scores of the CeHLS-D among the four response groups of internet use frequency (*F* = 35.50, *p* < .001) (Table 4). The effect size of the mean differences between the four groups was large ($\eta$^2^ = 0.19). A post-hoc test for group comparisons found that the mean scores on the CeHLS-D of the almost-every-day use group was significantly higher than those of the approximately-1-day-a-week and almost-no-use groups, and the CeHLS-D mean score of the several-days-a-week use group was significantly higher than that of the almost-no-use group. These findings indicate that the CeHLS-D had satisfactory known-groups validity.

**Table 4:** Known-groups validity by frequency of internet use groups

Group	*n*	Mean (SD)	*F* (*p*)	Post-hoc (Scheffe) test
Almost no use^a^	127	1.71 (1.19)	35.50 (< 0.001)	d > b > a, c > a
Approximately 1 day a week^b^	159	2.48 (0.83)		
Several days a week^c^	108	2.78 (0.79)		
Almost every day^d^	59	2.93 (0.83)		

SD, standard deviation

#### Floor and ceiling effects

Regarding the final CeHLS-D, participants achieved the lowest and highest scores for item 19 (Mean = 2.06, SD = 1.48) and item 16 (Mean = 3.20, SD = 1.09), respectively. The average scores for the total scale, factor 1, and factor 2 were 2.39 (SD = 1.04), 2.37 (SD = 1.04), and 2.58 (SD = 1.22), respectively (Table 5). The lowest floor effects of the participants for total score, factor 1, and factor 2 were 4.6%, 5.5%, and 3.8%, respectively; the corresponding ceiling effects were 2.6%, 4.6%, and 21.4%.

**Table 5:** Descriptive statistics of CeHLS-D items

Factor	Item	Abbreviated item description^a^	Mean (SD)
Factor 1	3	Thinking of search words	2.56 (1.22)
	6	Understanding medical terms	2.38 (1.25)
	7	Figuring out numeric medical examination values (e.g., HbA1c, fasting glucose)	2.63 (1.19)
	8	Appraising information credibility	2.32 (1.18)
	9	Distinguishing advertisements	2.41 (1.19)
	11	Trustworthiness of internet sources	2.19 (1.14)
	14	Filtering applicable information	2.13 (1.19)
Factor 2	15	Emailing	2.49 (1.56)
	16	Text messaging (e.g., KakaoTalk, WhatsApp)	3.20 (1.09)
	19	Sharing opinions on social media	2.06 (1.48)
Total scale of the CeHLS-D			2.39 (1.04)
Factor 1, cognitive actions for internet diabetes information			2.37 (1.04)
Factor 2, abilities of digital communication			2.58 (1.22)

^a^ Diabetes- and self-management-related information, terms, and sources

SD, standard deviation; Items are numbered as in Table 1.

## Discussion

This study developed the CeHLS-D under a comprehensive definition of eHealth literacy, which encompassed the attributes required for the social-media nature of the current digital environment. The CeHLS-D is the first condition-specific instrument for measuring eHealth literacy specifically in the context of diabetes. The Transactional eHealth Literacy Instrument [15] was developed in a population with chronic lung disease; however, there were no contents particularly relevant to the disease. So, the instrument was closer to a generic than to a condition-specific instrument.

Structural validity is defined as “the extent to which the structure of a multi-item instrument adequately reflects the hypothesized dimensionality of the construct being measured” [16]. Many psychometric studies of eHealth literacy instruments have only analyzed structural validity using EFA [6, 12, 55–65]. EFA was applied to reduce the numbers of items or to hypothesize the number of dimensions (factors) that the instrument had and which items were loaded on the factors. EFA was therefore considered to be not sufficiently adequate for structural validity [66]. A strength regarding the structural validity of the CeHLS-D was the application of a cross-validation approach. EFA yielded a two-factor solution for the CeHLS-D, and EGA under a network psychometric perspective supported that solution. CFA was then performed to verify whether the empirically hypothesized two-factor structure was fit for the actual data.

The CFA in this study yielded a correlation value of 0.778 between the two factors, which was considered a moderately strong correlation, and further exploration of the discriminant is required. Traditionally, the Fornell-Lacker criterion developed for marketing in 1981 (the average extracted variance of each construct > shared variance^2^) has been frequently applied to assess whether two latent variables (factors) in a measurement model of CFA are discriminant [67]. However, that criterion is criticized as lacking in discriminant detection [45]. In the present study, a recently recommended HTMT value was calculated, which confirmed the discriminant nature of the two-factor solution.

Cronbach’s alpha values may be higher when more items are included in an instrument [66]. The CeHLS-D is a relatively short instrument, and yet it achieved excellent Cronbach’s alpha values of 0.92 and 0.89 for factors 1 and 2, respectively. These values were similar or higher than those of other instruments with more items: 0.87–0.92 for the Transactional eHealth Literacy Instrument subscales [15], 0.77–0.86 for the eHealth Literacy Questionnaire subscales [14], 0.57–0.89 for the Digital Health Literacy Instrument subscales [12], and 0.52–0.81 for the eHealth Literacy Scale-Extended subscales [68]. Cronbach’s alpha has continually served as a dominant indicator of internal consistency. It is currently recommended that shifting to using ω would be a better alternative [69]. The ω values of the CeHLS-D subscales also demonstrated good internal consistency.

In this study, the measurement invariance of the CeHLS-D was satisfied across the gender, age, and glycemic control status groups. This means that the participants in the different groups recognized that not only the number of factors and patterns were the same, but also were the items loaded to the factors. The CeHLS-D can therefore be used in practice for people with type 2 diabetes regardless of gender, age, and glycemic control status.

Convergent validity is a test of the hypothesized relationship of scores on a focal instrument under study, with scores on a comparator measuring a similar construct. Here, the instrument used as a comparator was likely to be psychometrically satisfied. Regarding the convergent validity of existing eHealth literacy instruments, the measurement properties of their comparator instruments have not been reported for about 44% of studies, degrading the quality of convergent validity [7]. In the present study, the CeHLS-D exhibited satisfactory convergent validity with a moderate correlation using the psychometrically satisfied comparator of the DHLS to measure health literacy [30]. This was consistent with a study on the correlation between eHealth literacy measured using the eHealth Literacy Scale and health literacy measured using the Health Literacy Questionnaire subscales (*r* = .38–0.56) [35]. The CeHLS-D also had satisfactory known-groups validity. This was consistent with a study on a patient group that used the internet more frequently, which had had higher eHealth literacy than the other patient groups who used the internet several times a week or less [70].

There was a ceiling effect on the CeHLS-D subscale of the “abilities of digital communications.” This might attributable to the item of “text messaging (e.g., KakaoTalk, WhatsApp)”, which achieved the highest mean score among the items. In South Korea, 95% of the population owns a smartphone, 92% of those older than 13 years used a mobile messenger during the previous year, and around 99.1% of all mobile messenger users are utilizing KakaoTalk (a free mobile instant text messaging app) [71, 72]. Mobile text messaging, particularly using KakaoTalk, seems to be an essential communication method for South Koreans. Considering that, the item of “text messaging” might be a relatively easy skill for the participants of this study. It is therefore recommended to make that item more difficult or complicated in future studies. It also needs to be determined whether the ceiling effect was due to the cultural aspect of living in a society that is highly centered around digital communication.

All existing eHealth literacy instruments other than the eHealth Literacy Scale [6] were criticized for the instructions provided on how to respond to items not specifying the recall period, which may induce bias in the response items [7]. The instructions of the CeHLS-D classified the recall period as “at present.”

The CeHLS-D comprised 10 items with responses on a 5-point Likert-type scale. The number of items was much smaller than in the eHealth Literacy Scale-Extended (eHEALS-E) (20 items) [68], e-HLS (19 items) [11], DHLI (21 items) [12], eHLA (44 items) [13], eHLQ (35 items) [14], and TeHLI (18 items) [15]; but was larger than for the eHEALS (8 items) [6]. The CeHLS-D may therefore be feasible for use in practice and less burdensome to respondents.

### Limitations

This study had some limitations. First, the study design was cross-sectional, and the test–retest reliability—referring to the temporal stability for the same individuals on at least two occasions—of the CeHLS-D was therefore not assessed. Previous studies on the test–retest reliability of eHealth literacy instruments had several methodological limitations: insufficient sample sizes, no information on administration intervals, and/or using an inadequate reliability statistic [6, 55, 56, 73, 74]. It is therefore recommended to consider the limitations for further reliability tests of the CeHLS-D. Second, the criterion validity of the CeHLS-D was not tested because the patient-reported outcome measures (self-reporting instruments) almost always lack a gold standard, except when developing a short-version instrument using its corresponding long version as a gold standard for the criterion validity test [66]. In contrast, others insist that an expert opinion, physiological indicator, or clinical measure can be used as a gold standard for criterion validity [34]. If this is correct, it is recommended that further tests use an actual performance skill related to eHealth literacy as a criterion for the CeHLS-D. Third, the CeHLS-D was only psychometrically tested on Korean adults with type 2 diabetes, and so cross-cultural validation testing of the instrument is still needed.

### Implications for practice and research

With the rapid development of internet technology, individuals such as those with type 2 diabetes have come to seek health information on the internet and use the obtained information to make medical decisions [75]. However, these individuals have not equal abilities in evaluating whether information from the internet is effective or useful. In practice, health professionals have a responsibility to instruct patients about how to avoid obtaining conflicting or misleading internet diabetes information. To do so, they must identify patients with low eHealth literacy, and provide patients vulnerable to misleading or conflicting eHealth literacy information with methods and sources for trustworthy diabetes information in the internet. In such a situation, the CeHLS-D can be used for people with type 2 diabetes.

Diabetes self-management education is well known as an intervention that impacts self-management and glycemic control. The traditional delivery method of face-to-face diabetes education has recently been shifting to an internet- or app-based digital method. During the coronavirus disease 2019 pandemic, the importance of the digital delivery method came to the fore in clinical practice as a method for remote care and communication between health providers and patients. This suggests that internet- or app-based diabetes interventions or care should be tailored to eHealth literacy levels. The CeHLS-D can be used to assess eHealth literacy levels to develop tailored applications of internet-based diabetes interventions.

Even though the CeHLS-D is a population-specific instrument for type 2 diabetes, it may be applicable to patients with other chronic disease (e.g., hypertension), if some item phrases are adopted. For example, the phrase “information of diabetes and self-management” could be changed to “information of hypertension and self-management,” and the phrase “diabetes-related numeric medical examination values (e.g., HbA1c and fasting glucose)” changed to “hypertension-related numeric medical examination values (e.g., blood pressure and cholesterol).” To do so, a psychometric study on the adopted instrument (provisionally named CeHLS-hypertension) should be conducted on individuals diagnosed with hypertension.

## Conclusion

This study developed a new condition-specific eHealth literacy instrument for people with type 2 diabetes, designated as the CeHLS-D. The CeHLS-D comprises 10 items scored on a 5-point Likert scale; this brief instrument therefore has the strengths of being feasible for use in practice and being less burdensome to respondents. The CeHLS-D exhibited good psychometric properties of internal consistency, and content, structural, convergent, and known-groups validities. Its measurement invariance was also satisfied across gender, age, and glycemic control groups. The CeHLS-D can therefore be applied in research and practice to assess the eHealth literacy of people with type 2 diabetes. However, its test–retest reliability still needs to be evaluated, and a cross-cultural validity study is required among different languages and countries.

## Electronic supplementary material

Below is the link to the electronic supplementary material.

Supplementary Material 1

## Data Availability

The dataset used and/or analyzed during this study can be provided from the corresponding author on reasonable request.

## List of abbreviations

CeHLS-D: Condition-specific eHealth literacy scale for diabetes
CFA: Confirmatory factor analysis
CFI: Comparative fit index
DHLI: Digital health literacy instrument
EFA: Exploratory factor analysis
EGA: Exploratory graph analysis
eHealth literacy: Electronic health literacy
eHEALS: eHealth literacy scale
eHLQ: eHealth literacy questionnaire
e-HLS: e-Health literacy scale
HTMT: Heterotrait-monotrait ratio of correlation
I-CVI: Item-level content validity index
MGCFA: Multigroup confirmatory factor analysis
RMSEA: Root-mean-square error of approximation
SRMR: Standardized root-mean-square residual
TeHLI: Transactional eHealth literacy instrument
