# Supplementary material for: Development and psychometric evaluation of a new brief scale to measure eHealth literacy in people with type 2 diabetes
Source: BMC Nurs. 2022 Nov 4;21:297. doi: 10.1186/s12912-022-01062-2 (PMC9635185; doi:10.1186/s12912-022-01062-2)
Supplement: Supplementary file 1 — Supplementary Material 1 [file 12912_2022_1062_MOESM1_ESM.pdf]

Table S1. General information of interviewees

(*n* = 14)

Variable	*n* (%)
Gender	
Male	11 (78.6)
Female	3 (21.4)
Age, years	
40–49	2 (14.3)
50–59	7 (50.0)
60–69	4 (28.6)
≥70	1 (7.1)
Marital status	
Married/living with partner	13 (92.9)
Unmarried	1 (7.1)
Employed	
Yes	12 (85.7)
No	2 (14.3)
Education	
High school	3 (21.4)
College and above	11 (78.6)
Treatment regimen	
Oral hypoglycemic agent	13 (92.9)
Oral hypoglycemic agent + insulin	1 (7.1)
HbA1c	
Controlled, HbA1c ≤ 6.5%	1 (7.1)
Uncontrolled, HbA1c > 6.5%	13 (92.9)
	Mean±SD
Duration of disease, years	12.7±8.2

HbA1c, glycated hemoglobin A1c

Table S2. General characteristics of the study participants

Variable	Total sample (N= 453)	Subsample 1 (*n* = 231)	Subsample 2 (*n* = 222)
	n (%)	*n* (%)	*n* (%)
Gender			
Male	293 (64.7)	144 (62.3)	117 (50.6)
Female	160 (35.3)	87 (37.7)	114 (49.4)
Age, years			
<30	10 (2.2)	5 (2.2)	5 (2.3)
30–39	19 (4.2)	10 (4.3)	9 (4.0)
40–49	67 (14.8)	33 (14.3)	34 (15.3)
50–59	165 (36.4)	83 (35.9)	82 (37.0)
60–69	144 (31.8)	77 (33.3)	67 (30.1)
≥70	48 (10.6)	23 (10.0)	25 (11.3)
Employed			
Yes	300 (66.2)	153 (66.2)	147 (66.2)
No	153 (33.8)	78 (33.8)	75 (33.8)
Education			
Middle school and below	55 (12.1)	25 (7.8)	30 (13.5)
High school	188 (41.5)	89 (38.5)	99 (44.6)
College and above	210 (46.4)	117 (50.6)	93 (41.9)
Treatment regimen			
Oral hypoglycemic agent	354 (78.1)	186 (80.5)	168 (75.7)
Insulin	4 (0.9)	3 (1.3)	1 (05)
Oral hypoglycemic agent + insulin	95 (21.0)	42 (18.2)	53 (23.9)
HbA1c			
Controlled, HbA1c ≤6.5%	153 (33.8)	80 (34.6)	73 (32.9)
Uncontrolled, HbA1c >6.5%	300 (66.2)	151 (65.4)	149 (67.1)
	Mean±SD	Mean±SD	Mean±SD
Duration of disease, years	8.9±7.3	8.7±7.0	9.2±7.5

HbA1c, glycated hemoglobin A1c; SD, standard deviation.
